# Circulation of chikungunya virus East/Central/South African lineage in Rio de Janeiro, Brazil

**DOI:** 10.1371/journal.pone.0217871

**Published:** 2019-06-11

**Authors:** Joilson Xavier, Marta Giovanetti, Vagner Fonseca, Julien Thézé, Tiago Gräf, Allison Fabri, Jaqueline Goes de Jesus, Marcos Cesar Lima de Mendonça, Cintia Damasceno dos Santos Rodrigues, Maria Angélica Mares-Guia, Carolina Cardoso dos Santos, Stephane Fraga de Oliveira Tosta, Darlan Candido, Rita Maria Ribeiro Nogueira, André Luiz de Abreu, Wanderson Kleber Oliveira, Carlos F. Campelo de Albuquerque, Alexandre Chieppe, Tulio de Oliveira, Patrícia Brasil, Guilherme Calvet, Patrícia Carvalho Sequeira, Nuno Rodrigues Faria, Ana Maria Bispo de Filippis, Luiz Carlos Junior Alcantara

**Affiliations:** 1 Laboratório de Patologia Experimental, Instituto Gonçalo Moniz/Fiocruz, Salvador, Bahia, Brazil; 2 Laboratório de Flavivírus, Instituto Oswaldo Cruz, Fundação Oswaldo Cruz, Rio de Janeiro, Brazil; 3 Laboratório de Genética Celular e Molecular, Instituto de Ciências Biológicas, Universidade Federal de Minas Gerais, Belo Horizonte, Minas Gerais, Brazil; 4 KwaZulu-Natal Research Innovation and Sequencing Platform (KRISP), College of Health Sciences, University of KwaZulu-Natal, Durban, South Africa; 5 Department of Zoology, University of Oxford, Oxford, United Kingdom; 6 Departamento de Genética, Instituto de Biologia, Universidade Federal do Rio de Janeiro, Brazil; 7 Secretaria de Vigilância em Saúde, Ministério da Saúde, Brasília, Distrito Federal, Brazil; 8 Organização Pan-Americana da Saúde/Organização Mundial da Saúde, Brasília, Distrito Federal, Brazil; 9 Superintendência de Vigilância do Estado, Rio de Janeiro, Brazil; 10 Instituto Nacional de Infectologia Evandro Chagas, Rio de Janeiro, Brazil; Fundacao Oswaldo Cruz Instituto Rene Rachou, BRAZIL

## Abstract

The emergence of chikungunya virus (CHIKV) has raised serious concerns due to the virus’ rapid dissemination into new geographic areas and the clinical features associated with infection. To better understand CHIKV dynamics in Rio de Janeiro, we generated 11 near-complete genomes by means of real-time portable nanopore sequencing of virus isolates obtained directly from clinical samples. To better understand CHIKV dynamics in Rio de Janeiro, we generated 11 near-complete genomes by means of real-time portable nanopore sequencing of virus isolates obtained directly from clinical samples. Our phylogenetic reconstructions indicated the circulation of the East-Central-South-African (ECSA) lineage in Rio de Janeiro. Time-measured phylogenetic analysis combined with CHIKV notified case numbers revealed the ECSA lineage was introduced in Rio de Janeiro around June 2015 (95% Bayesian credible interval: May to July 2015) indicating the virus was circulating unnoticed for 5 months before the first reports of CHIKV autochthonous transmissions in Rio de Janeiro, in November 2015. These findings reinforce that continued genomic surveillance strategies are needed to assist in the monitoring and understanding of arbovirus epidemics, which might help to attenuate public health impact of infectious diseases.

## Introduction

Chikungunya virus (CHIKV) infections have been reported worldwide since the virus was first isolated in Tanzania in 1953 [1]. Over 70 CHIKV epidemics have been reported around the world between 1959 and 2016 [2]. Only in the Americas, more than 1 million cases were notified in 2017, with Brazil reporting 185,593 cases [3, 4].

CHIKV is an Alphavirus from the *Togaviridae* family that is transmitted via the bite of infected *Aaedes aegypti* and *Aedes albopictus* mosquitoes [5, 6]. CHIKV infection results in a dengue-like febrile disease, whose acute phase is characterized by high fever, macular or maculopapular rash, backache, headache, fatigue, myalgia, and polyarthralgia [7]. Patients may also develop a later chronic condition, with persistent or relapse symptoms of arthropathy [7]. As the majority of CHIKV infected patients are symptomatic with varying degrees of joint or musculoskeletal pains and require long-term treatment, CHIKV represents a serious burden inflected on both public health and economic sector [7–9].

There are four CHIKV genotypes (or lineages), which have been named based on their geographical distribution: (i) the West African; (ii) the East/Central/South African (ECSA); (iii) the Asian; and (iv) the Indian Ocean Lineage (IOL), which emerged from the ECSA lineage between 2005 and 2006 [10–12]. Studies have shown that several IOL strains presented a series of mutations including the E1-A226V adaptive mutation that confers an increased replication rate in *Aedes albopictus*, thus enhancing viral transmission potential [11, 13, 14].

Local transmission of the Asian lineage was detected in Brazil for the first time in September 2014 in Oiapoque municipality, in the northern region [15]. Seven days later, a new cluster of CHIKV infections was notified in the city of Feira de Santana, Bahia state, north-eastern Brazil [15]. Sequencing of three isolates revealed those infections in Bahia were caused by the ECSA lineage, signalling its emergence and establishment in the country [15]. Since then, the ECSA genotype has been detected in several other Brazilian states in the north-eastern, south-eastern and northern regions, exerting a serious threat to public health, especially because the ECSA lineage is associated with higher symptomatic to asymptomatic ratio compared with the Asian lineage circulating in the Caribbean [16–21].

CHIKV infections in Brazil accounted for 277,882 and 185,593 suspected cases in 2016 and 2017, respectively [22]. In 2018, the country registered 87,687 suspected cases, most of which in south-eastern Brazil (52,966; 60.4%) [4]. In 2016, Rio de Janeiro state reported 18,516 CHIKV suspected cases until epidemiological week 52 [22]. The epidemic persisted and, in 2018, Rio de Janeiro state reported 39,725 cases, with the municipalities of Rio de Janeiro (10,062 cases) and São Gonçalo (6,261 cases) municipalities being among the most affected by the epidemic [4].

The first cases of CHIKV autochthonous transmissions in Rio de Janeiro were registered in late 2015, although there is some serological evidence of imported CHIKV infections acquired abroad in 2010 [23–25]. Limited molecular and evolutionary information are available on the CHIKV epidemic in Rio de Janeiro state, although sequencing of two partial genomes revealed that the ECSA lineage was the strain circulating in the state during the 2016 epidemic [26, 27].

The state of Rio de Janeiro is located in the south-eastern region of Brazil and it registered an influx of 1,6 million airline passengers and 12,710 international and domestic flights, during the Olympic Games in August 2016 [28]. Moreover, the state, whose capital city is the second biggest of Brazil, is an important hub for busyness, tourism and it is well connected to the main metropolitan regions in the country. Nevertheless, there is limited information about the genomic epidemiology of CHIKV circulating in Rio de Janeiro from genomic surveillance studies. The shortage of complete genomic sequences available impairs our understanding of the CHIKV introduction and establishment in the region. Thus, to better understand the chikungunya outbreak in Rio de Janeiro, we generated 11 near-complete genomes collected from 2016 to 2018 using portable nanopore sequencing and present a genomic epidemiology that sheds new light on the introduction and establishment of ECSA lineage in Rio de Janeiro.

## Materials and methods

### Ethics statement

This research was reviewed and approved by the Ethical Committee of the Pan American World Health Organization (No. PAHO-2016-08-0029) and the Brazilian Ministry of Health (MoH) as part of the arbovirus genomic surveillance efforts within the terms of Resolution 510/2016 of CONEP (Comissão Nacional de Ética em Pesquisa, Ministério da Saúde; National Ethical Committee for Research, Ministry of Health). Human samples were previously obtained for routine diagnostic purposes from adult patients visiting local public clinics in Rio de Janeiro. Residual anonymized clinical diagnostic samples, with no or minimal risk to patients, were provided for research and surveillance purposes within the terms of Resolution 510/2016 of CONEP. Processing of human samples was approved and the need for participants consent was waived by the Institutional Review Board from the Fundação Oswaldo Cruz/Instituto Oswaldo Cruz (CEP/CAAE: 90249218.6.1001.5248; approval number 2.998.362).

### Diagnostic assays

Under the scope of the ZIBRA project, an itinerant virus surveillance project supported by the Brazilian Ministry of Health (zibraproject.org), we analysed 102 samples from patients presenting symptoms compatible with arbovirus infection. Those samples were collected by the Central Laboratory of Public Health that sought the diagnostic services provided by the National Reference Laboratory for Epidemiological Surveillance of Arbovirus in the Laboratory of Flavivirus at the Oswaldo Cruz Institute (IOC) from the Oswaldo Cruz Foundation (Fiocruz), Rio de Janeiro, Brazil.

Viral nucleic acid extraction was performed using the Magmax Pathogen RNA/DNA kit and the KingFisher Flex Purification System (Thermo Fisher), used for automated magnetic-particle processing for RNA. Molecular diagnostic was performed by RT-qPCR using the protocol from [29] with the following modifications: use of GoTaq Probe 1-Step RT-qPCR System kit (Promega) on a LineGene 9660 (Bioer) machine. All procedures were conducted in biological safety cabinets located in physically separated areas. Negative controls were used in all reactions.

### Synthesis of cDNA and multiplex tiling PCR

All 102 positive samples were submitted to a cDNA synthesis protocol [30] using ProtoScript II First Strand cDNA Synthesis Kit. Then, a multiplex tiling PCR was conducted using Q5 High Fidelity Hot-Start DNA Polymerase (New England Biolabs) and CHIKV sequencing primers scheme (primers are divided into two separated pools, A and B) designed using Primal Scheme (http://primal.zibraproject.org) [31]. The thermocycling conditions involved 45 cycles, and reaction conditions were previously reported in [31].

### Library preparation and nanopore sequencing

Amplicons were purified using 1x AMPure XP Beads (Beckman Coulter) and cleaned-up PCR products concentrations were measured using Qubit dsDNA HS Assay Kit on a Qubit 3.0 fluorimeter (ThermoFisher). DNA library preparation was performed on 11 selected samples (selection based on DNA concentration after clean-up being > 4ng/μL) using the Ligation Sequencing Kit (Oxford Nanopore Technologies) and Native Barcoding Expansion 1–12 kit (Oxford Nanopore Technologies), whose reactions conditions have already been described [31], with the following modifications: The same sample is added to both sequencing primers pools (A and B, separated tubes) during multiplex tiling PCR. After PCR, each pool is purified, and its DNA concentration is quantified using Qubit. Then, both pools (A and B) are joined in a single tube (taking in consideration the DNA concentrations of each pool), and one barcode was used per sample in order to maximize the number of samples per flow cell. Sequencing library was generated from the barcoded products using the Genomic DNA Sequencing Kit SQK-MAP007/SQK-LSK208 (Oxford Nanopore Technologies). Sequencing library was loaded onto a R9.4 flow cell (Oxford Nanopore Technologies). Sequencing was performed for 48 hours on MinION device, although data acquisition can be performed from 4h to 72h. Acquisition of the final consensus sequences was performed following a previously published protocol [31].

### Phylogenetic and bayesian analysis

The 11 new genomic sequences reported in this study were initially submitted to a genotyping analysis performed by Genome Detective virus tool online (https://www.genomedetective.com/). New sequences were aligned to 229 complete or almost complete CHIKV genomic sequences (>10,000 bp), retrieved from NCBI in November 2018 and covering all four existing lineages. We also included 6 sequences from a recent outbreak in Brazilian Amazon region [16], and 16 sequences from Rio de Janeiro recently published [32]. Alignment was performed using MAFFT online program [33]. The complete dataset was assessed for presence of phylogenetic signal by applying the likelihood mapping analysis implemented in the IQ-TREE 1.6.8 software (http://www.iqtree.org) [34]. A maximum likelihood (ML) phylogeny was reconstructed from the concatenated dataset (n = 240) using IQ-TREE 1.6.8 software under the HKY nucleotide substitution model with 4 gamma categories (HKY+G4) which was inferred in jModelTest (https://github.com/ddarriba/jmodeltest2) as the best fitting model [35].

From the generated maximum likelihood (ML) phylogeny using the concatenated dataset we selected all ECSA taxa from Brazil (ECSA-BR dataset) (n = 59). In order to investigate the temporal signal in our CHIKV-ECSA dataset, we regressed root-to-tip genetic distances from this ML tree against sample collection dates using TempEst v 1.5.1 (http://tree.bio.ed.ac.uk) [36].

The ML phylogeny was used as a starting tree for Bayesian time-scaled phylogenetic analysis using BEAST 1.10.2 (http://beast.community/index.html) [37]. We employed a stringent model selection analysis using both path-sampling (PS) and stepping stone (SS) procedures to estimate the most appropriate molecular clock model for the Bayesian phylogenetic analysis [38]. We tested: a) the strict molecular clock model, which assumes a single rate across all phylogeny branches, and b) the more flexible uncorrelated relaxed molecular clock model with a lognormal rate distribution (UCLN) [39]. Both SS and PS estimators indicated the strict molecular clock (Bayes Factor = 4.3) as the best fitted model to the dataset under analysis. Besides, we have used the he HKY+G4 codon partitioned (CP)1+2,3 substitution model and the Bayesian SkyGrid coalescent model of population size and growth [39, 40]. We computed MCMC (Markov chain Monte Carlo) duplicate runs of 50 million states each, sampling every 5.000 steps for the ECSA-BR dataset. Convergence of MCMC chains was checked using Tracer v.1.7.1 [41]. Maximum clade trees were summarized from the MCMC samples using TreeAnnotator (http://beast.community/index.html) after discarding 10% as burn-in.

### Epidemiological data assembly

Data of weekly notified CHIKV cases in Brazil were supplied by Brazilian Ministry of Health and were plotted using the R software version 3.5.1 (The R Foundation).

## Results

To better understand the diversity of CHIKV in some of most affected municipalities from Rio de Janeiro, we generated 11 CHIKV near-complete genomes (coverage range 62%-83%, mean = 73%) from serum samples using a nanopore sequencing approach [31]. This genome coverage obtained is considered sufficient to perform phylogenetic inferences, according to a study that showed the occurrence of a decrease in phylogenetic accuracy when genome coverage is reduced from 40% to 20% [42].

Mean threshold cycle value of the generated sequences was 14.86. Most of the isolates are from patients that reside in the municipality of Rio de Janeiro (n = 6), and the other five isolates are from four different neighbouring municipalities. We did not selected isolates features, such as sampling location and collection date, since, at random, only 11 samples proved to be suitable for sequencing (they had enough DNA concentration after purification, >4ng/μL). Of the sequenced samples, 6 are from female and 5 from male patients. Ten samples are from over 40 years old patients, and one sample is from a 26 years old female. None of the patients had travelled to previous epidemic areas as indicated by the epidemiological data from the local surveillance service. Sequencing statistics and epidemiological details of the sequences generated here are available in Tables 1 and 2.

**Table 1:** Epidemiological data for the sequenced samples.

ID	Sample	Collection date	Sex	Age	Municipality	State
RJ39	Serum	2016-02-19	F	74	Rio de Janeiro	RJ
RJ74	Serum	2016-04-05	M	65	Rio de Janeiro	RJ
RJ83	Serum	2016-04-27	F	88	Rio de Janeiro	RJ
RJ94	Serum	2016-05-02	M	53	Rio de Janeiro	RJ
RJ95	Serum	2016-05-06	F	53	Rio de Janeiro	RJ
RJ96	Serum	2016-05-10	M	54	Rio de Janeiro	RJ
RJ105	Serum	2016-04-19	F	67	São João de Meriti	RJ
RJ111	Serum	2016-04-05	M	57	Mesquita	RJ
RJ125	Serum	2017-03-07	M	70	São Gonçalo	RJ
RJ127	Serum	2017-03-09	F	26	Niteroi	RJ
RJ137	Serum	2018-02-18	F	49	São João De Meriti	RJ

ID = study identifier; Municipality = Municipality of residence; State = RJ-Rio de Janeiro; F = Female; M = Male.

**Table 2:** Sequencing statistics for the 11 new obtained sequences.

ID	Accession number	Reads	Bases	Coverage (%)	QC	Ct
RJ39	MK244635	58,566	22,587,905	65.7552	1006	14.6
RJ74	MK244632	103,884	39,905,972	62.3518	1006	10.8
RJ83	MK244634	56,876	22,567,764	74.0857	1006	14.1
RJ94	MK244636	64,688	26,009,860	82.5432	1006	14.8
RJ95	MK244633	65,235	25,887,105	74.5852	1006	15.6
RJ96	MK244637	82,622	32,005,376	70.8009	1006	14.7
RJ105	MK244639	51,939	21,750,261	72.5025	1006	15.2
RJ111	MK244638	163,802	63,997,867	76.7863	1006	19.8
RJ125	MK244640	68,806	28,017,200	79.8002	1006	13.3
RJ127	MK244641	73,636	28,692,714	70.1998	1006	15.5
RJ137	MK244642	126,118	48,272,106	75.8805	1006	15.1

ID = study identifier; Accession number = NCBI accession number; QC = Quality control of a flow cell-number of available pores; Ct = RT-qPCR quantification cycle threshold value.

To investigate the phylogenetic relationship of CHIKV in the southeast region of Brazil we estimated Maximum Likelihood (ML) for a dataset comprising 240 international sequences of the four CHIKV lineages. We also investigated the Brazilian ECSA clade in more detail using both ML and Bayesian molecular clock approaches, where we included the recently published ECSA isolates from Brazilian Amazon region [16].

Our ML phylogeny revealed that the newly generated CHIKV isolates from Rio de Janeiro belong to the ECSA lineage, clustering with other Brazilian isolates from the north-eastern region that also belong to this lineage (S1 Fig). These results were confirmed by a genotyping analysis implemented in the Genome Detective virus online tool (https://www.genomedetective.com/).

**Fig 1:**
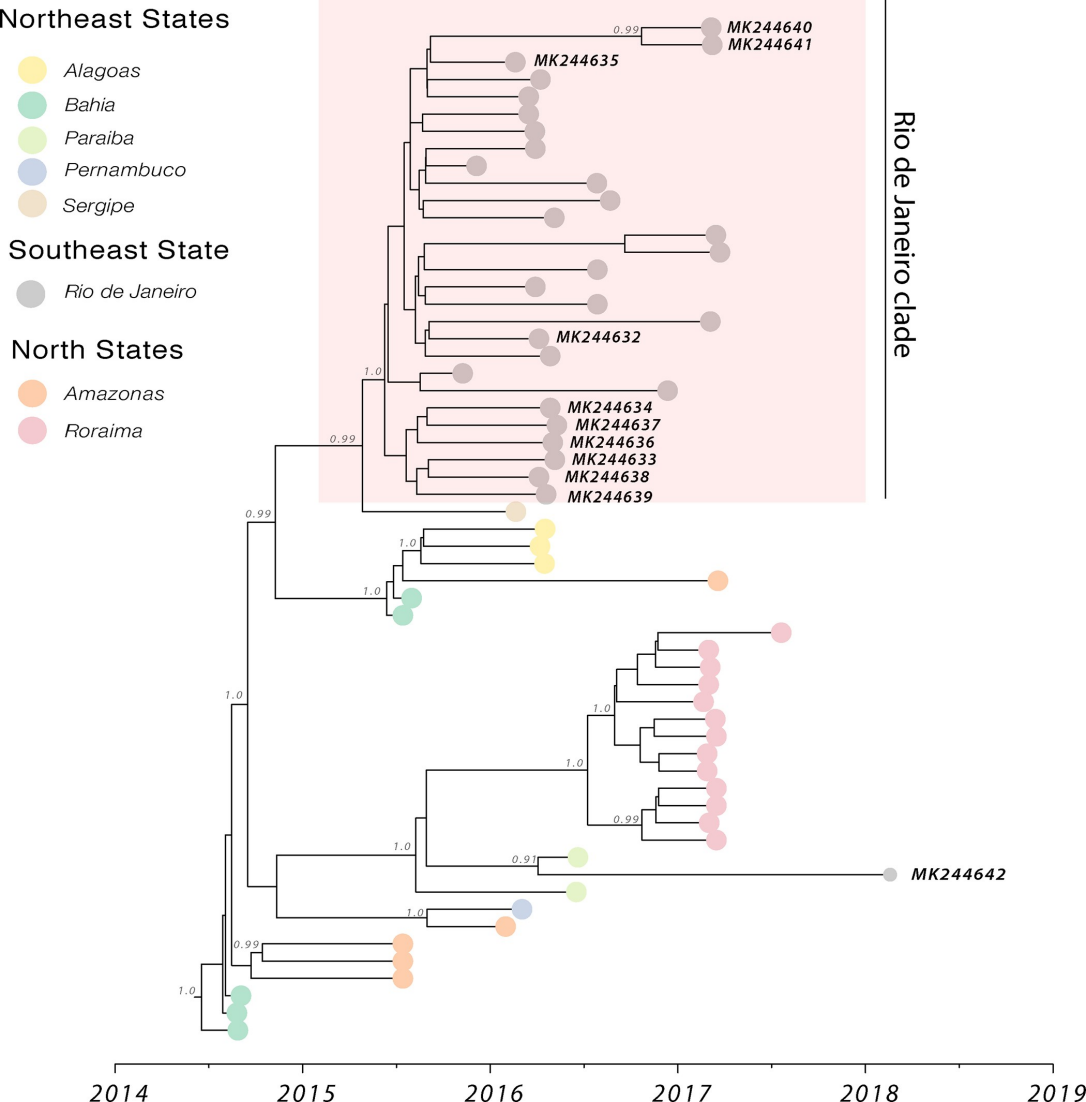
Molecular clock phylogeny of the CHIKV circulating in Rio de Janeiro state. Molecular clock phylogeny obtained using 11 new CHIKV near-complete genomic sequences from the 2016–2018 epidemic in Rio de Janeiro, including 48 publicly available Brazilian CHIKV-ECSA lineage sequences. Numbers along branches represent clade posterior probability >0.90. Colours represent different locations.

**Fig 2:**
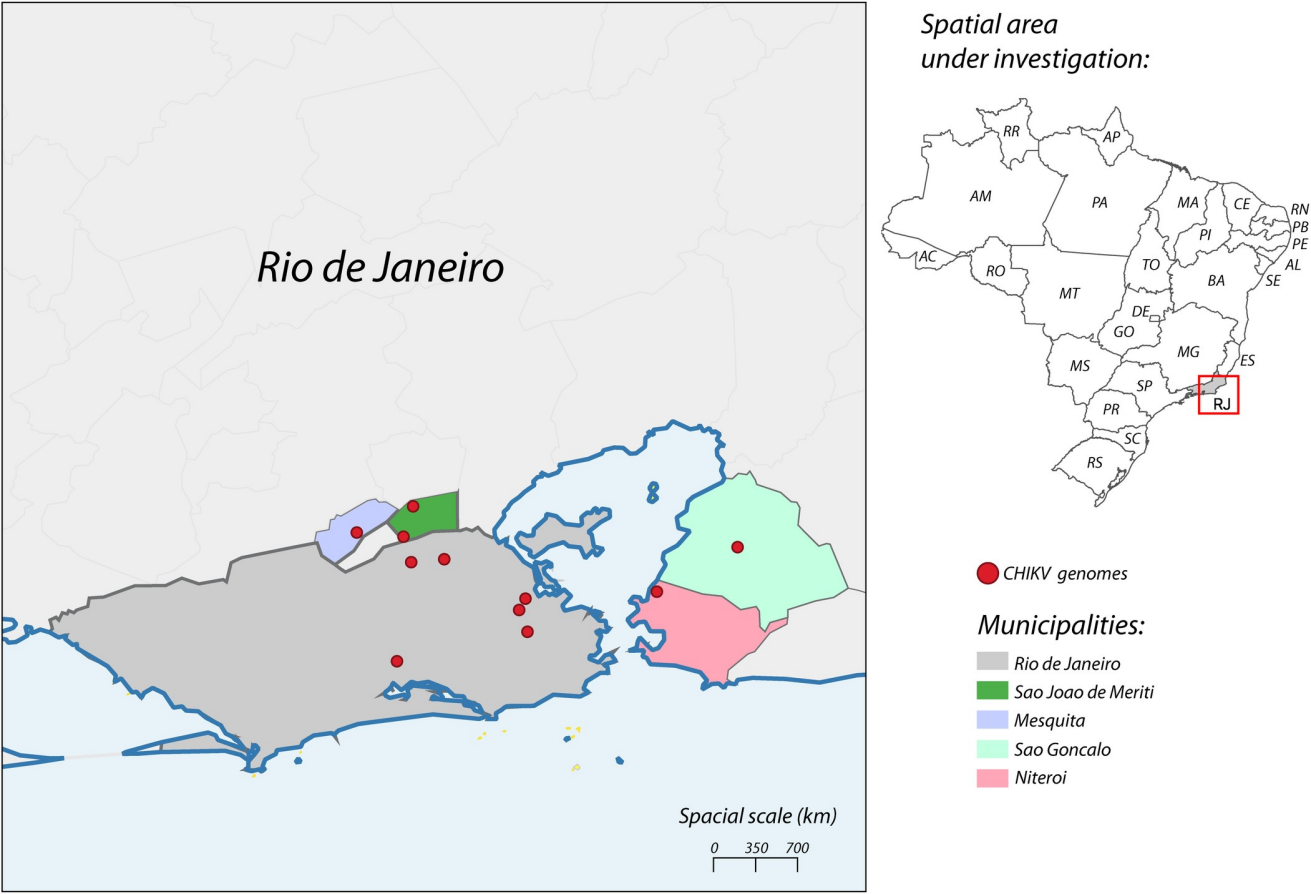
Map of the state of Rio de Janeiro. **The state of Rio de Janeiro is located in south-eastern region of Brazil and its municipalities, where samples from this study were collected, are coloured in the map (see legend).** Red circles indicate sampling locations of the isolates generated in this study. Map was generated by QGIS v3 software.

To investigate the time of introduction of the ECSA lineage in the state of Rio de Janeiro, we performed a sampling time-scaled phylogenetic analysis using a Bayesian Markov Chain Monte Carlo (MCMC) framework [37]. A regression of genetic divergence from root to tip against sampling dates confirmed sufficient temporal signal (r2 = 0.68). Our Bayesian time-scaled phylogenies showed that ten (90.9%) genomes generated in this study formed a well-supported clade (posterior probability support = 1.0) comprising isolates from Rio de Janeiro state, sampled between 2015–2017; while only one isolate from 2018 (isolate RJ137, accession number: MK244642) was placed in a different cluster with an isolate from Paraíba state (northeast Brazil) sampled in 2016 (accession number: KY704954) (Fig 1). Moreover, an isolate from Sergipe state (accession number: KY055011), northeast Brazil, and sampled from in 2016, closely clustered to the Rio de Janeiro clade (Fig 1). The observed clustering of the 2016–2017 CHIKV isolates reported here probably reflects geographical proximity of the municipalities where samples were collected in Rio de Janeiro (Fig 2). Although the RJ105 and RJ137 isolates are both from the same municipality of São João de Meriti, they have different sampling collection dates, 2016 and 2018 respectively. We identified 10 amino acid substitutions that were present only in the RJ137 isolate when compared to the other 10 isolates generated in this study (S2 Table). The presence of these unique mutations in the RJ137 isolate is consistent with its latest sampling date, indicating that this isolate had more time to undergo genetic change and its clustering with an isolate (KY704954) from a northeast state might represent a second event of viral introduction in Rio de Janeiro.

The date of the most recent common ancestor (tMRCA) of the Rio Janeiro clade was estimated to June 2015 (95% Bayesian credible interval: May to July 2015) (Fig 1). This estimation suggests the ECSA lineage circulated unnoticed for approximately 5 months before the first reports of CHIKV autochthonous transmissions in Rio de Janeiro, in November 2015 [23, 24].

**Fig 3:**
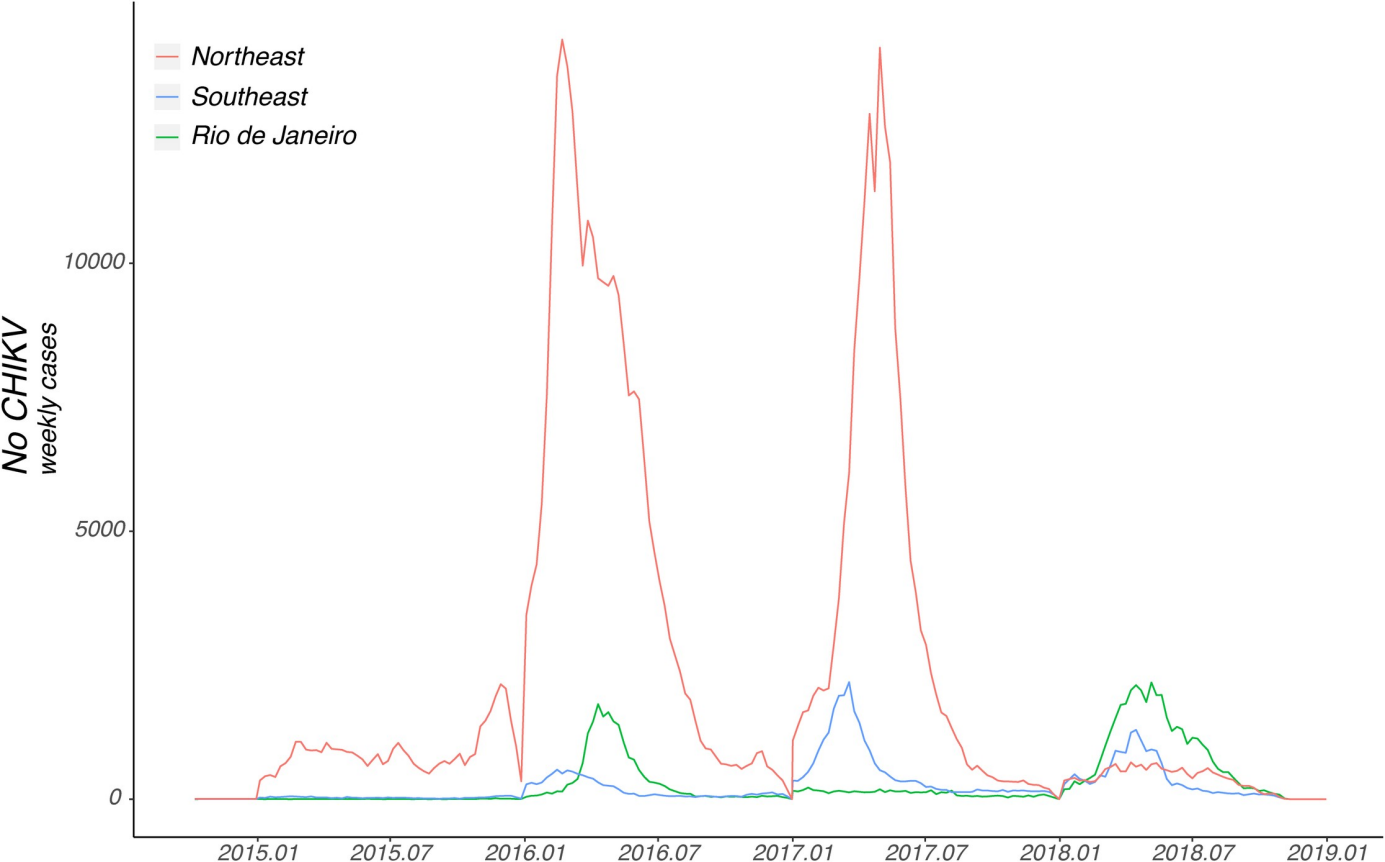
Weekly notified Chikungunya fever cases in the north-eastern and south-eastern regions of Brazil, 2015–2018. Number of chikungunya fever cases by epidemiological week registered in the north-eastern and south-eastern regions of Brazil between 2015 and 2018. Epidemic curves are coloured according to geographical region (see legend). Number of cases from the state of Rio de Janeiro alone is represented by the green curve and it is not computed in the curve from southeast. Chart was generated by R software.

Fig 3 shows the number of chikungunya cases weekly notified in the north-eastern region, the south-eastern (without computing numbers from Rio de Janeiro state), and in Rio de Janeiro state alone. While the north-eastern region presented intense epidemic waves in early 2016 and 2017, reporting 239,714 and 141,312 cases, respectively, the south-eastern region reported a lower number of cases in those years, accounting for 28,556 and 32,314 notified cases in 2016 and 2017, respectively.

Simultaneous circulation of other arbovirus, such as dengue (DENV) and zika (ZIKV) occurs in both northeast and southeast regions of Brazil, and surveillance reports from the Brazilian Ministry of Health provide the annual number of dengue e zika cases reported in those regions [4, 22, 43]. The southeast region registered > 92,000 zika cases in 2016, followed by a notable decrease in the number of cases in 2017 and 2018 (chart A in S2 Fig). The same dynamics was observed for the number of dengue cases in that region (chart B in S2 Fig). Similarly, the northeast region reported in 2016 >300,000 and >75,000 cases of dengue and zika, respectively (S2 Fig). In the following years of 2017 and 2018 a marked decrease has been observed in the number of cases of these infections in the northeast region.

Fig 3 also shows an increase in chikungunya number of cases registered in Rio de Janeiro in 2016 and then a notable reduction in 2017, followed by an increase in CHIKV transmission in 2018 accounting for 39,208 reported cases until epidemiological week 44. Rio de Janeiro’s epidemic curve also shows that the state alone accounted for 66.8% and 69.7% of the cumulative cases registered in the south-eastern region in 2016 and 2018, respectively. While a reduction in the number of cases was observed in the north-eastern region in 2018, the south-eastern region kept experiencing increased CHIKV transmission (Fig 3). In general, CHIKV cases peaked in the early months of the represented years, consistent with previous virus dynamics predictions (Fig 3) [15, 44].

We found that epidemic waves from Rio de Janeiro state and the north-eastern region displayed a synchronicity during the period from late 2015 to early months of 2016 (Fig 3), and there is a high human mobility between the two regions [28]. From combined epidemiological and genetic data, we found that the northeast Brazil is the most likely source location of the ECSA-lineage strain that was circulating unnoticed during the 2016 epidemic in the state of Rio de Janeiro.

## Discussion

In this study, by performing real-time portable nanopore sequencing, we generated 11 new CHIKV near-complete genomic sequences from 2016–2018 collected in several municipalities in the Rio de Janeiro state. The generated genomic data allowed us to estimate the introduction date of the ECSA lineage in Rio de Janeiro to June 2015, suggesting an undetected circulation of the virus for 5 months before the first reports of CHIKV transmission in Rio de Janeiro [23, 24]. According to the Ministry of Health epidemiological bulletin, CHIKV autochthony in Rio de Janeiro was reported in the 47^th^ epidemiological week of 2015, around November 2015. Prior to that, only imported cases had been registered. Our estimates indicate a more recent introduction event of the ECSA lineage in Rio de Janeiro compared to a recently published study [32]. In our analysis we used a larger and updated dataset including recently published 18 new CHIKV ECSA sequences from northern region of Brazil. The substantial difference (around 17 months) between Souza et al. (2019) [32] estimates and ours might reflect evolutionary models choice and the analyzed dataset. Nevertheless, our results are in agreement with Nunes et al. (2015) [15] and the epidemiological data reported by the Brazilian health system [23, 24].

Moreover, our data suggests that the circulation of the CHIKV-ECSA lineage in Rio de Janeiro may have resulted from at least two separate introductions events from the north-eastern region, where this lineage was first detected in late 2014 [15]. The first event is indicated by the Rio de Janeiro clade which was dated to mid-2014 and consists of 28 isolates, including the 10 sequences generated in this study. The second introduction event, in turn, is represented by the RJ137 isolate, sampled in 2018, which formed a cluster with one isolate (KY704954) sampled in 2016 from a north-eastern state, and this cluster was dated to mid-2015.

Our results corroborate previous estimates of the introduction date of the CHIVK ECSA lineage in the north-eastern region of Brazil in mid-2014 [15]. After its introduction, the risk of the ECSA lineage to spread from Bahia to other Brazilian states was estimated to be higher in the northeast and southeast regions due to abundance of competent vectors and climate conditions in those geographical areas [15, 44]. CHIKV ECSA lineage spread to other north-eastern states, such as Paraíba, Sergipe, Pernambuco and Alagoas states, causing large outbreaks until it arrived in the southeast region, where this lineage caused a total of 25,245 suspected cases in 2016 alone [17, 18, 21, 22, 32]. The ECSA lineage also reached the Brazilian Amazon region in 2017 [16].

This pattern of arbovirus cryptic transmission prior to first official reports have been observed before for dengue and zika virus epidemics [45, 46]. Genetic and epidemiological analysis indicated the northern of Brazil acted as a source region for dengue, or as stepping-stone spot for the dissemination of arbovirus to other areas of the country, which might have been influenced by the increased human mobility and vector suitability [45]. Similarly, zika virus strains from the 2015–2016 epidemic in Brazil circulated cryptically in the north-eastern region and, from there, disseminated to other Brazilian states and countries before its first detection in the Americas [46]. Climatic data suggests that both the northern and north-eastern regions are able to sustain year-round transmission of mosquito-borne viruses, making them putative sources of transmission [46].

We performed protein alignments of our obtained sequences to investigate the presence of the A226V (E1 protein) or the L210Q (E2 protein) substitutions, however we did not observe in our samples any of those mutations associated with increased CHIKV transmission in *Ae*. *albopictus* mosquitos [11, 13, 14]. However, future investigation of the fitness, viral infectivity and evolution of CHIKV in mosquito populations, together with continued genomic surveillance, will determine whether the upsurge in the number of cases in Rio in 2018 was due to the acquisition of specific mutations that increase replication rates in local *Aedes* spp. mosquitoes.

Genome sequencing has become a powerful tool for tracking virus transmission. The analysis of full or near full length genomes maximize the usefulness of molecular clock models to infer time-scaled phylogenetic trees. These models can statistically describe the relationship between viruses genetic distances and sampling time to infer phylogenies, where branch lengths represent time, and this natural timescale of months or years can be used as a reference for directly comparing evolutionary rate with known historical events [36]. Hence, by analysing heterochronous datasets with samples collected in different time points and/or locations, time-scaled phylogenies become a powerful tool to describe trends in epidemic spread [47]. This approach has been used in genomic epidemiology studies of arbovirus during epidemics in Brazil [15–17]. Sequencing and phylogenetic analysis of 54 genomes of ZIKV sampled during the 2015–2017 epidemic provided estimates of the date of introduction and the period of cryptic circulation of the virus in north-eastern Brazil, as well as the date of dissemination of the virus to other countries in the Americas [46]. Later on, between 2016–2017, the same phylogenetic approach was employed to investigate the largest Yellow Fever outbreak that Brazil has witnessed since 1928 [30]. By analyzing 64 new yellow fever virus genomes the virus transmission pattern was revealed to have origin in non-human primates, rejecting the hypothesis of urban transmissions.

Together, our results indicate CHIKV ECSA lineage might have spread to Rio de Janeiro from the north-eastern region of Brazil and shed light on the epidemiological dynamics of the virus in urban areas of the Rio de Janeiro, where the virus remained undetected by several months before reporting of the first local transmission cases in that state. In conclusion, our study shows that genomic data generated by real time portable sequencing technology can be employed to assist public health laboratories in monitoring and understanding the diversity of circulating mosquito-borne viruses.

## Supporting information

S1 Fig Maximum likelihood phylogeny of chikungunya virus. (TIF)

S2 Fig Annual number of dengue and zika cases registered in northeast ad southeast regions of Brazil, 2016–2018. (TIF)

S1 Table Accession number list of the reference sequences retrieve from NCBI. (XLSX)

S2 Table Nucleotide substitutions observed in the genome of RJ137 isolate. (PDF)
