# Supplementary figures and images for: Circulation of chikungunya virus East/Central/South African lineage in Rio de Janeiro, Brazil
Source: PLoS One. 2019 Jun 11;14(6):e0217871. doi: 10.1371/journal.pone.0217871 (PMC6559644; doi:10.1371/journal.pone.0217871)

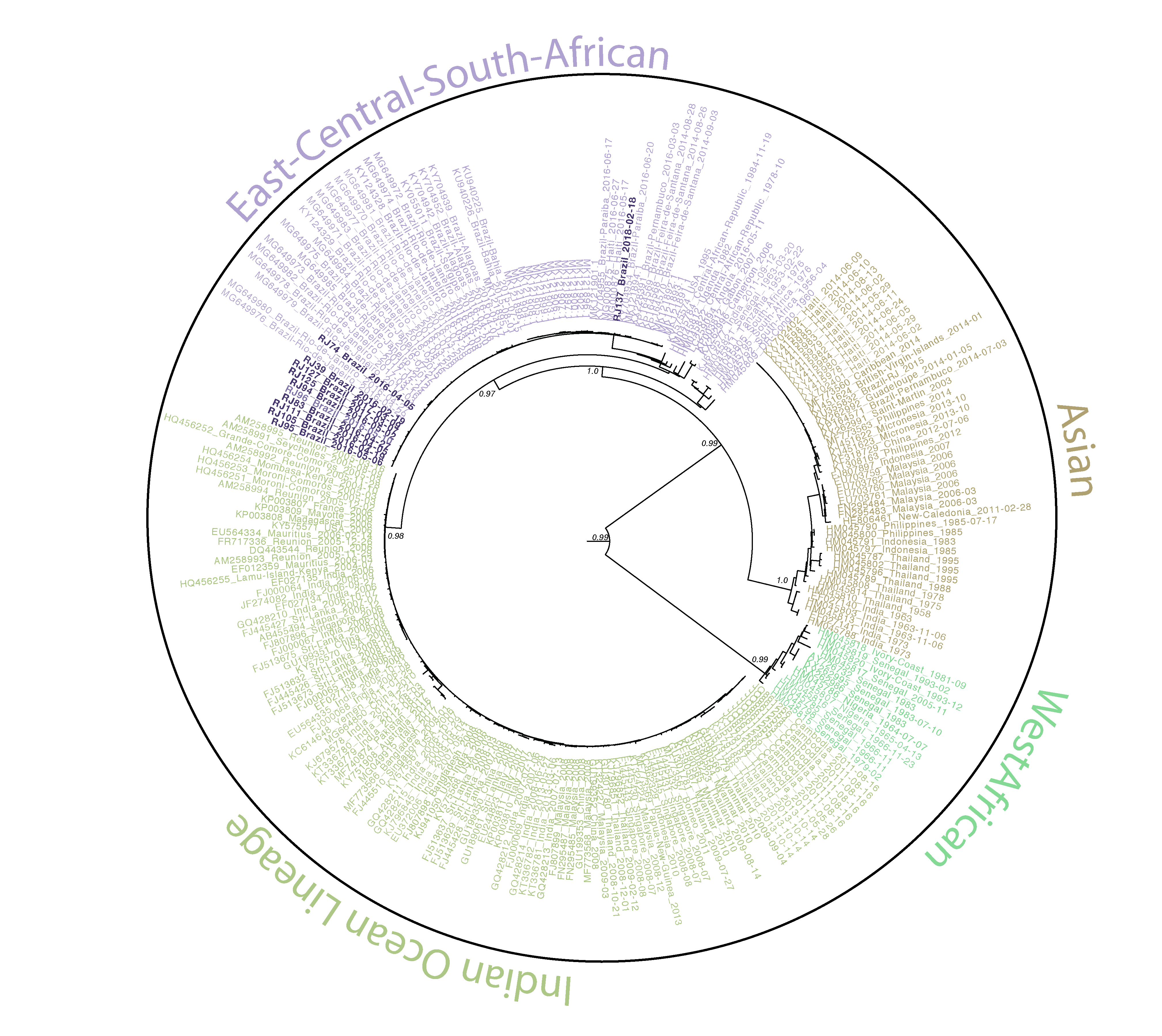

Supplement: S1 Fig — (TIF) [file pone.0217871.s001.tif]

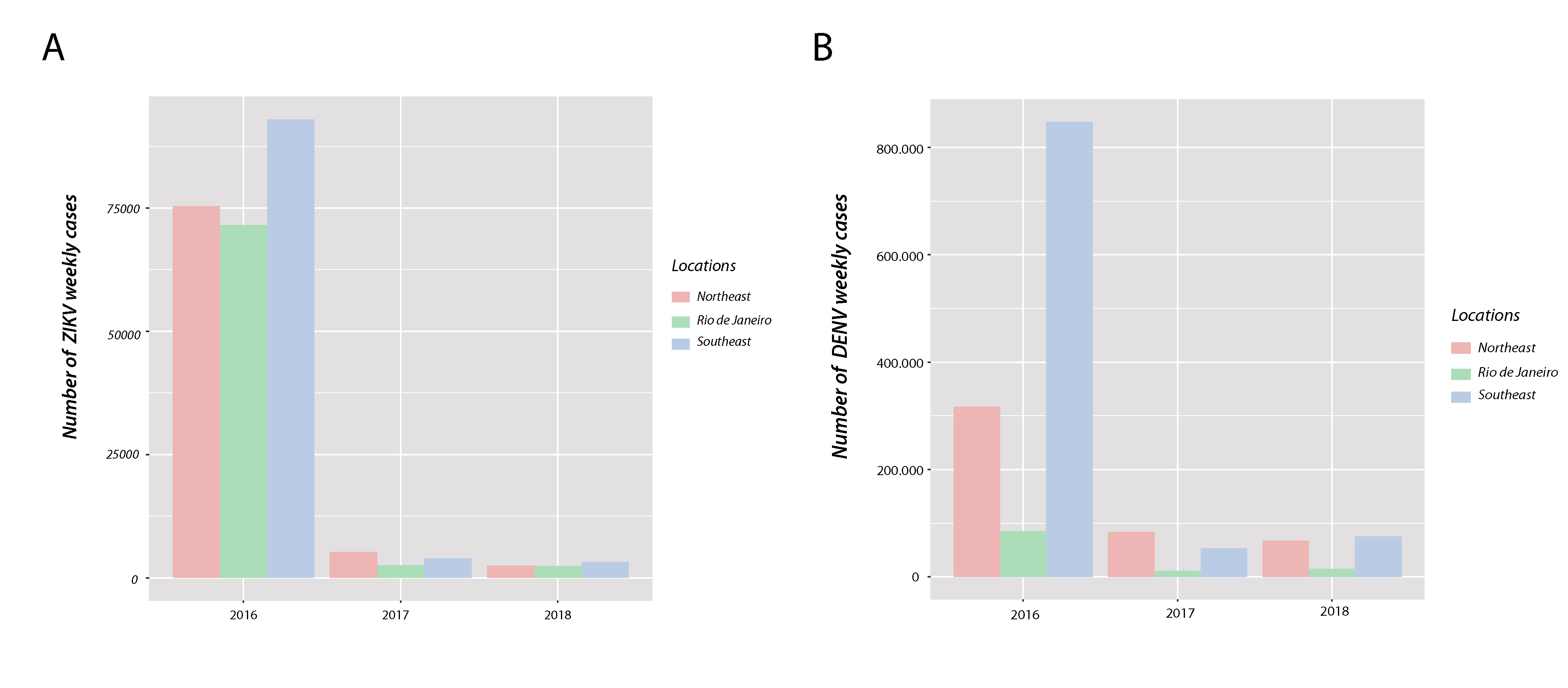

Supplement: S2 Fig — (TIF) [file pone.0217871.s002.tif]
