# Supplementary material for: Circulation of chikungunya virus East/Central/South African lineage in Rio de Janeiro, Brazil
Source: PLoS One. 2019 Jun 11;14(6):e0217871. doi: 10.1371/journal.pone.0217871 (PMC6559644; doi:10.1371/journal.pone.0217871)
Supplement: S2 Table — (PDF) [file pone.0217871.s004.pdf]

S2 Table. Nucleotide substitutions observed in the genome of the RJ137 isolate.

Genome nucleotide position	Amino acid
C1069T	Y
C1912T	F
G2735C	P
G3250A	T
G3415A	M
G5996A	D
C6670T	C
A7107C	S
G9074A	H
C10625A	K
